# Two conserved amino acids differentiate the biology of high‐risk and low‐risk HPV E5 proteins

**DOI:** 10.1002/jmv.27829

**Published:** 2022-05-14

**Authors:** Sawali R. Sudarshan, Richard Schlegel, Xuefeng Liu

**Affiliations:** ^1^ Department of Pathology Center for Cell Reprogramming, Georgetown University Medical School Washington District of Columbia USA; ^2^ Department of Pathology Wexner Medical Center, The James Comprehensive Cancer Center, The Ohio State University Columbus Ohio USA

**Keywords:** cellular effect, gene expression, human papillomavirus, oncogenesis, oncoproteins, virus classification

## Abstract

The high‐risk alpha human papillomaviruses (HPVs) are responsible for 99% of cervical cancers. While the biological functions of the HPV E6 and E7 oncoproteins are well‐characterized, the function of E5 has remained elusive. Here, we examined gene expression changes induced by E5 proteins from high‐risk HPV‐16 and low‐risk HPV‐6b in multiple pools of primary human keratinocytes. Surprisingly, microarray analysis revealed that over 700 genes were significantly regulated by HPV‐6b E5, while only 25 genes were consistently and significantly regulated by HPV‐16 E5 in three biological replicates. However, we observed that more than thousand genes were altered in individual sample compared with vector. The gene expression profile induced by 16E5 in primary genital keratinocytes was very different from what has been previously published using immortalized HaCaT cells. Genes altered by HPV‐16 E5 were unaffected by HPV‐6b E5. Our data demonstrate that E5 proteins from the high‐ and low‐risk HPVs have different functions in the HPV‐host cell. Interestingly, conversion of two amino acids in HPV‐16 E5 to the low‐risk HPV‐6b sequence eliminated the induction of high‐risk related cellular genes.

## 1 INTRODUCTION

1.1

The human papillomavirus (HPV)‐16 E5 (16E5) protein is a weak transforming protein that resides in membranes of the endoplasmic reticulum (ER) and modulates cell growth and viral replication.^1^, ^2^, ^3^, ^4^, ^5^, ^6^ 16E5 can self‐associate both in vitro and in vivo and form oligomers by hydrophobic interactions.^7^, ^8^ E5 proteins are classified into E5α, E5β, E5γ, and E5δ using predictive approaches of the biochemical characteristics and protein evolution, where those of high‐risk HPVs fall in the E5α category and those of low‐risk fall in to the E5β, E5γ, or E5δ families.^9^ E5 proteins are small hydrophobic transmembrane proteins containing three hydrophobic trans‐membrane domains (TMD1‐3), based on molecular prediction and modeling analysis.^9^ E5 is postulated to expand the initial population of HPV‐infected basal cells, perhaps by enhancing EGFR activation.^4^, ^10^, ^11^ While 16E5 alone cannot immortalize human primary cells, it can induce anchorage‐independent growth of immortalized rodent cells in soft agar^12^, ^13^, ^14^, ^15^, ^16^, ^17^ and enhance cell immortalization by E6/E7.^18^, ^19^, ^20^ In addition, estrogen‐treated transgenic mice expressing 16E5 as well as E6 and E7 develop a larger number of tumors than mice expressing E6 and E7 alone.^11^, ^13^ Given the importance of E5 in BPV, the natural question is whether these transforming properties are shared by E5s of the human papillomavirus. Phylogenetic studies indicated that E5s of low‐risk HPVs cluster together with each other and separately from E5s of high‐risk E5s.^9^ The correlation of phylogeny with cancer risk suggested that HPV‐16 E5 might also contribute to tumorigenesis.^9^, ^11^, ^21^, ^22^

The main oncoproteins of HPV‐16 are E6 and E7, which are both necessary and sufficient for cell immortalization. E5 is neither necessary nor sufficient for immortalization. Besides E2, E5 is one of the other proteins that is assumed to be disrupted during viral integration.^23^, ^24^ Estimations for the percentage of HPV‐induced cervical cancers that have integrated DNA – and therefore potentially no E5—varies greatly, from 15% to 86%.^25^, ^26^, ^27^ One study estimated that only 60% of HPV‐16 induced cervical cancers might express E5.^4^, ^28^

For several reasons, E5 is considered the third oncoprotein of HPV. First of all, the lack of E5 at later stages of malignant transformation does not mean that early E5 expression is not essential in establishing a successful and persistent infection (the precursor to dysplasia and cancer). It has been suggested that E5 helps to expand the initial population of HPV‐infected basal cells, perhaps by enhancing EGFR activation.^4^, ^10^, ^12^, ^17^, ^29^, ^30^ Second, while E5 is present in all high‐risk viruses, many low‐risk types either lack an E5 ORF altogether or lack a translation start codon.^9^, ^21^ Finally, E5 is able to enhance the transformation of cells by E6 and E7 in‐vivo. For example, it was shown that estrogen‐treated transgenic mice expressing HPV‐16 E5 in addition to E6 and E7 developed a larger number of tumors than mice expressing E6 and E7 alone.^11^, ^31^ How E5 actually causes these observed phenotypes is still under debate, although there are several possibilities, including EGFR activation, activation of c‐jun and c‐fos, binding of v‐ATPase, disruption of gap junctions, immune evasion, formation of koilocytes, and binding of nuclear transport proteins.^4^, ^32^, ^33^, ^34^

We previously reported that 16E5, as well as HPV‐6b E5 (6bE5), induce koilocytosis in collaboration with E6.^33^ The mechanism behind these 16E5‐induced phenotypes is unknown. However, the ability of 16E5 to bind several cellular proteins, including the 16‐kDa subunit of the vacuolar H^+^‐ATPase,^35^, ^36^, ^37^ BAP31,^38^ HLA,^39^, ^40^, ^41^, ^42^, ^43^ ErbB4,^44^ calnexin,^43^ and karyopherin β3^32^ might account for some of its biological activities. Little is known about the biologic functions and cellular partners of E5 proteins of low‐risk HPVs. Here, we examined gene expression changes induced by E5 proteins from high‐risk HPV‐16 and low‐risk HPV‐6b in multiple pools of primary human keratinocytes. Our microarray analysis revealed that over 700 genes were significantly regulated by HPV‐6b E5, while only 25 genes were consistently and significantly regulated by HPV‐16 E5 in three biological replicates. Genes altered by HPV‐16 E5 were unaffected by HPV‐6b E5. Conversion of two amino acids in HPV‐16 E5 to the low‐risk HPV‐6b sequence eliminated the induction of high‐risk related cellular genes. Our data demonstrate that E5 proteins from the high‐ and low‐risk HPVs have different functions in the HPV‐host cell.

## 2 MATERIALS AND METHODS

### 2.1 Constructs

E5 mutant constructs were generated by Celtek Biosciences. All constructs have an N‐terminal AU1 tag (DTYRYI). A Kozak sequence (CTCGAG) was also included 5′ of the start codon. For cloning purposes, constructs were built with EcoRI (5′), XhoI (5′), BamH1 (3′) and SalI (3′) restriction sites flanking the E5 open reading frame. EcoRI and BamH1 sites were used to clone the construct into the pLXSN vector for stable expression (Clontech).

### 2.2 Cells and cell culture

Human foreskin keratinocytes (HFKs) were prepared from human foreskins donated by Georgetown University Hospital. HFKs were maintained in Keratinocyte Serum‐Free Media (Invitrogen), supplemented with 50 g/ml bovine pituitary hormone, 26 ng/ml recombinant epidermal growth factor, and 10 g/ml gentamycin (KGM). All cells were maintained using T75 or T175 flasks. RNA or western blot lysates were collected from 100 mm tissue culture plates, all from BD Falcon.

### 2.3 Cell transduction

5 × 10^6^ SD3443 retroviral packaging cells were plated per 100 mm dish overnight in Dulbecco's modified Eagle's medium (DMEM) complete. After 24 h, media was replaced with 5 ml serum‐free DMEM and plates were treated with 25 μM chloroquine for at least 15 min. Cells were transfected with 4 μg of DNA using Lipofectamine Plus transfection reagent (Invitrogen) per the manufacturer's protocol for retrovirus packaging. After 4 h, 5 ml of complete DMEM with 20% fetal bovine serum (FBS) was added to the plate. The next day, the media was replaced with 5 ml fresh DMEM complete with 10% FBS. After 24 h, retrovirus was collected by harvesting the supernatant and filtering it through a 0.22 mm filter (Millipore) to remove cell particulates and ensure sterility. Retrovirus was either used fresh or stored at −80°C until needed.

To transduce cells, 1.5 ml retroviral stock supplemented with 1.5 μl polybrene was added to cells in T75 flasks at 40%–60% confluency. Cells were incubated with the retrovirus on a gentle rocker at 37°C. After 2 h, the retrovirus was removed and replaced with media appropriate to the cell type. Cells were allowed to grow to approximately 80%, which occurred within 1–3 days. For cell selection, geneticin (G418) (Invitrogen) at a concentration of 75–100 μg/ml was used and selection was maintained until all the cells in the control (uninfected) flask died.

### 2.4 Immunoflorescence

24 h after transfection (COS‐1 cells) or plating (for stably‐expressing cells such as HFKs and HECs), cells were washed with phosphate‐buffered saline (PBS) and fixed with 4% (w/v) paraformaldehyde. After 10' incubation on ice and subsequent 15' incubation at room temperature, cells were washed four times with PBS. Cells were then permeabilized with 0.1% (w/v) saponin for 10 min, washed 2 times with PBS, and blocked for 20 min in a humid box with P‐GelS (PBS with 0.2% gelatin and 0.1% saponin) and 20% normal donkey serum. After three PBS washes, cells were covered with primary antibody for 1 h in the humid box, followed by another three washes. Primary antibodies used were rabbit polyclonal anti‐AU1 (1:1500 dilution, Covance) and rabbit polyclonal anti‐calnexin (1:75 dilution, Santa Cruz). Cells were incubated with a 1:400 dilution of Alexa‐flour secondary antibody for 1 h at room temperature. After another three PBS washes, cells were washed with PBS containing 2% gelatin. Then, nuclei were stained with 0.5 mg/ml Hoeschst stain for 3 min at room temperature. Coverslips were then removed and inverted over slides with 30 μl mounting media (Invitrogen) and allowed to rest at room temperature for several hours until the mounting media hardened. Slides were stored at 4°C overnight and viewed the next day using a Zeiss Axioskop microscope (Carl Zeiss, Inc.). Cells were imaged using a 63X objective, Hammamutsu CCD camera, and Openlab 3.0.7 software.

### 2.5 Cell lysis and protein concentration

For direct western, whole‐cell lysates were made by plating cells on 100 mm dishes (BD Falcon) and allowing them to grow to 80% confluence. Plates were washed with cold PBS, and cells scraped in 300 μl of two times Laemlli buffer. Lysates were kept on ice, then boiled for 10' at 110°C, allowed to cool for 2 min, and frozen on dry ice. Before protein assay, lysates were thawed in a 37°C water bath. Before loading, up to 45 μl of sample (40–60 μg protein) was mixed with a volume of β‐mercaptoethanol (Sigma‐Aldrich) equal to 10% of the final loading volume. For immunoprecipitation, cells were scraped instead with 1.2 ml radioimmunoprecipitation assay (RIPA) buffer with 12 μl protease inhibitor cocktail set 1 (Calbiochem, 100X stock) and frozen on dry ice. Before protein assay, lysates were thawed in a 37°C water bath, DNA was sheared with a 23 G needle, and lysates were spun down at 2 K rpm. Protein concentration for both lysates was determined using the BioRad D_c_ Protein Assay (Bio‐Rad Laboratories) per the manufacturer's protocol.

### 2.6 Immunoprecipitation

Equal amounts of protein (up to 600 μg) per sample were added to 40 μl Protein A Plus beads (Pierce). After washing with 1 ml PBS, beads were rotated for 90' end‐to‐end with antibody. After being spun down for 1' at 2k rpm, beads were washed with 1 ml cold RIPA buffer with protease inhibitors, followed by an additional 5' rotation and 1' centrifugation. This was repeated two times more, followed by three consecutive washes with PBS (no rotation). Beads were pelleted and then resuspended in 47 μl two times Laemmli with 10% βme. No βme was added if reducing conditions were not to be used (as for E5 dimerization studies). After 20 min in a 37°C water bath, beads were boiled for 6 min at 110°C before being frozen on dry ice. Before gel loading, samples were thawed in a 37°C water bath. Antibodies used for E5 immunoprecipitation included rabbit and mouse anti‐AU1 (BABCO/Covance), used at 4 μl/tube (~4  μg).

### 2.7 Western blot

Samples were electrophoretically separated on Tris‐Glycine gels (Invitrogen) and transferred to polyvinylidene difluoride (PVDF) membranes (Millipore). Membrane was blocked for 30' in either PBS with 5% nonfat dry milk or in wash buffer with 2% bovine serum albumin, depending on the antibody. Primary antibody anti‐AU1 was left overnight at 4°C on a rocker. ß‐actin (Sigma‐Aldrich) at a final dilution of 1:10 000, served as the loading control. Membrans was washed two times for 15' with either PBS + .05% Tween or wash buffer (Fisher Scientific). Membranes were then probed with a secondary antibody, anti‐mouse IgG.

### 2.8 RNA extraction and generation of cDNA

RNA was harvested from 100 mm tissue culture dishes (BD Falcon) at 80% confluence using 1 ml TRIzol Reagent according to manufacturer's protocol. DNAse treatment was done according to the manufacturer's protocol for the Rnaqueous‐4PCR Kit. RETROScript kit (Ambion) was used to perform reverse‐transcriptase polymerase chain reaction (RT‐PCR). RNA was denatured for three minutes at 80°C with Oligod(T) and Random Hexamers. This was followed by the reverse‐transcriptase step consisting of 60 min at 45°C and 10 min at 92°C. cDNA samples were diluted between 25 and 75 ng/μl (with a 10–100x dilution for GAPDH) and stored at −20°C until needed for PCR or for real‐time PCR.

### 2.9 Microarray

cDNA microarray analysis was performed on HFKs that were retrovirally infected and selected for HPV‐16 E5, HPV‐6b E5, or pLXSN. Each sample from E5‐expressing keratinocytes was run against the pLXSN vector in a two‐color Agilent whole human genome slide with a 4 × 44 K format. For each E5, there were a total of six arrays, consisting of three biological replicates run in twice for dye swapping. RNAs were extracted and sent to MOGene, LC for microarray analysis. RNA was amplified using the Agilent Low Input Linear Amplification kit (Agilent Technologies), and then labeled with either cyanine‐5 or cyanine‐3 using the ULS aRNA Fluorescent Labeling Kit (Kreatech Biotechnology) according to manufacturer's instructions. 825 ng each of labeled c‐DNA was hybridized overnight at 65°C in an ozone‐free room to protect the label. All washes and hybridization conditions followed were consistent with the Agilent processing manual (protocol version 4.0). Arrays were scanned using Agilent scanner (G2505B) and extracted using the Agilent Feature Extraction software (Agilent Technologies). Analysis of data by MOGene was done using the GeneSpring software (Agilent). The Bioinformatics and Biostatistics Shared Resource at the Georgetown University Lombardi Comprehensive Cancer Center performed pre‐processing and differential analysis, including calculating average fold change and p‐values, using Rosetta Resolver (Rosetta Biosoftware, Microsoft).

### 2.10 PCR for E5 expression

cDNA was prepared as previously described and then used for PCR. This involved a preliminary denaturation step at 94°C for 4 min, followed by 30 cycles of: 30 s at 94°C, 30 s at 55°C, and 45 s at 72°C. This was followed by a final extension step of 10 min at 72°C. E5‐specific and GAPDH primers used include: HPV‐16 E5 and mutants‐Forward: 5′‐GCTGGCCTGCTTTCTGCTGT‐3′‐Reverse: 5′‐CCTAAAGGCAGAGGCTGCTG‐3′; HPV‐6b E5‐Forward: 5′‐TGTACACATCTGTGCTAGTACT‐3′‐Reverse: 5′‐GGACAGTAACACACAAGTA‐3′; HPV‐6b E5 Mutant (6bYI)‐Forward: 5′‐GGCACCACATCAACCTTTAT‐3′‐Reverse: 5′‐TATAGACGATGAACTCGCTG‐3′; GAPDH forward: 5′‐TCTCCTCTGACTTCAACAGC‐3′‐reverse: 5′‐GAAATGAGCTTGACAAAGTG‐3′.

PCR products were run using a 1.2% gel on a Flash Gel System (Lonza), and photographed under ultraviolet light.

### 2.11 Quantitative real time PCR

cDNA was prepared as previously described and then used for real‐time PCR. GAPDH served as the control. Real‐time reactions were 20 μl and contained 0.8 μl cDNA at 75 ng/μl, 10 μl 2x Bio‐Rad IQ SYBR Green Supermix (Bio‐Rad Laboratories), 0.125 20 μM primer mix (forward and reverse primers), and 9.08 μl dH_2_O. Primers using these conditions were ordered from RealTimePrimers.com and include: MED26‐F: 5′‐AGC ATC CAT GAC CTG AAG AG‐3′ and ‐R: 5′‐AAG CTC TCT GGA CTC CCA CT‐3′; UBE2E1‐F: 5′‐GCA AAC CGA GAA AGA AAC AA‐3′ and ‐R: 5′‐GGC CCT AGA ATG GTT GAT CT‐3′; GPR135‐F: 5′‐AGG GCT ACC GGA CTA GGA AT‐3′ and ‐R: 5′‐TTA GGC TGT TTG GTC ACT GC‐3′; CDK2NC‐F: 5′‐AAT GGA TTT GGA AGG ACT GC‐3′ and ‐R: 5′‐CAG CTT GAA ACT CCA GCA AA‐3′; MMP9‐F: 5'‐CTC TGG AGG TTC GAC GTG‐3′ and ‐R: 5′‐GTC CAC CTG GTT CAA CTC AC‐3′; PLA2G4C‐F: 5′‐ATC GAT TTA CCC GAC AGG AG‐3′ and ‐R: 5′‐GGG TAG TGT CCC TTC TTC CA‐3′; SERPINA3‐F: 5′‐CTC AGT CTG CTG GAC AGG TT‐3′ and ‐R: 5′‐TGA GTA TCT TGG GGG TCA AA‐3′; ICAM1‐F: 5′‐TTT TCT ATC GGC ACA AAA GC‐3′ and ‐R: 5′‐AAT GCA AAC AGG ACA AGA GG‐3′. Three biological replicates for each sample were run in triplicate on a 96‐well plate and spun down for 5′ at a low RPM. Reactions were annealed and analyzed using a Bio‐Rad iCycler and accompanying software (Bio‐Rad Laboratories).

## 3 RESULTS AND DISCUSSION

**Figure 1:**
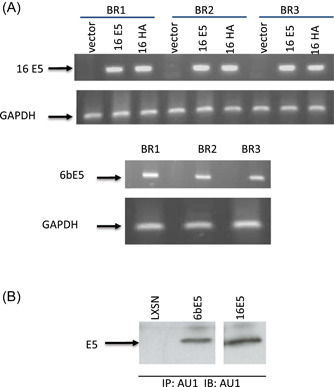
Expression of E5 genes in human foreskin keratincutes (HFKs). (A) RT‐PCR. Specific primer sets for 16E5 and 6bE5 were used for RT‐PCR assays on RNA samples from three biological replicates, GAPDH was used as internal control. LXSN was vector control. (B) Immunuprecipitation and western blot (IP/WB). RIPA lysates from E5 transduced HFKs were immunoprecipitated and blotted with monoclonal antibody against AU1. LXSN was vector control. RT‐PCR, reverse‐transcription polymerase chain reaction

To define the biological activities of E5 proteins, we analyzed E5‐induced changes in gene expression in primary human genital keratinocytes. The only previous microarray analysis examining 16E5 expressing cells was performed in HaCat cells, a spontaneously immortalized adult trunk keratinocyte cell line with mutant p53.^19^ 16E5 expression in these cells induces high levels of apoptosis, requiring the use of an inducible promoter. Consequently, analysis is temporally limited following the induction of 16E5 and is potentially confounded by the apoptotic and genetic changes in these cells. Rather than using immortalized cells as a target, we chose to use primary genital keratinocytes to more closely mimic the effect of high‐risk HPV‐16 E5 and low‐risk HPV6B E5 on cellular gene expression in vivo. To ensure that these changes were reproducible and physiologically relevant, we performed the microarray assays in triplicate. We first verified that all E5s expressed at similar levels using RT‐PCR (Figure 1A) and IP/WB (Figure 1B). Surprisingly, we found that 16E5 consistently regulated fewer than 25 genes across all arrays conducted (>1.5 fold change in each array, *p*‐value < 0.01) (Table 1A), even though individual array had more than thousand genes altered by 16E5 compared with LXSN (Supporting Information Table). Interestingly, we also found that all of these consistently regulated genes were downregulated. These genes were functionally grouped using the Gene Ontology Biological Process (BP) database^45^ as shown in Table 1B. In stark contrast, statistical analysis of the six 6bE5‐vs‐LXSN arrays revealed more than 750 genes that were changed consistently (both upward and downward) across different HFK donors (>1.5 fold change and *p* value < 0.01 for all six arrays). In addition, these genes were changed with far greater fold change than those discovered for 16E5 (range of −13.9 to +326) (Supporting Information Table). The top 25 up and downregulated genes for 6bE5 are documented in Table 2A,B. All ~760 genes regulated by 6bE5 were also submitted for functional grouping according to the Gene ontology BP database (Table 2C). We noted interesting numbers of genes overlapped in arrays, overlapped gens from 16E5 and 6bE5 arrays were 47, this was similar between 16E5 and 16HA (49 genes), while we noticed 237 genes were overlapped in 6bE5 and 16HA. These data further demonstrated that two amino acids in 16E5 contributed to different biological functions of HR and LR HPV E5 proteins.

**Table 1:** Microarray analysis reveals 16E5‐induced alteration of the keratinocyte gene expression profile.

(A) Less than 25 genes were found to be consistently changed (>1.5 fold in each array, *p* < 0.01) in 16E5‐expressing cells as compared to LXSN‐expressing cells.			
Primary sequence name	Accession #	*p* Value	Fold change
AHNAK	NM_001620	0.00	−1.65
FLJ00399	AK090477	0.01	−1.70
GPR135	NM_022571	0.00	−1.71
FLJ20802	AK000809	0.00	−1.71
SPRED1	NM_152594	0.00	−1.73
NR3C1	U25029	0.00	−1.76
LOC440345	AK123481	0.00	−1.76
LOC440248	NM_199045	0.00	−1.80
BX090412	BX090412	0.00	−1.90
APP	CK8188527	0.00	−1.94
PRO1073	AF001542	0.00	−1.95
PTGS2	NM_000963	0.00	−1.99
PITPNC1	AK094724	0.00	−2.03
LOC440345	AK123481	0.00	−2.08
ADH1A	BX647987	0.00	−2.08
CYP3A7	NM_000765	0.00	−2.18
PCDH9	BC008476	0.01	−2.28
LOC283970	XM_934220	0.00	−2.60
UBE2E1	NM_003341	0.00	−2.73
LOC150759	AK057596	0.00	−3.14
CD47	NM_001777	0.00	−3.20
MED26	BC030138	0.00	−6.11

**(B) Genes were classified according to enriched gene ontology terms**.			
**GO Class ID**	**Definitions**	**Counts**	**Fractions**
GO:0008152	metabolism	6	28.60%
GO:0006629	lipid metabolism	6	28.60%
GO:0009058	biosynthesis	3	14.30%
GO:0007275	development	3	14.30%
GO:0007165	signal transduction	1	4.80%
GO:0007154	cell communication	1	4.80%
GO:0040007	growth	1	4.80%
**Total**		**21**	**100.00%**

*Note*: Microarray was performed on three different donor pools of primary foreskin keratinocytes which were stably transduced with 16E5 or LXSN. Dye swap was performed for each replicate. (A) Less than 25 genes were found to be consistently changed (>1.5 fold in each array, *p* < 0.01) in 16E5‐expressing cells as compared to LXSN‐expressing cells. (B) Genes were classified according to enriched gene ontology terms. Significantly represented (*p* < 0.01) biological processes are shown.

**Table 2:** Microarray analysis reveals gene expression changes induced by 6bE5.

(A) Top 25 genes (by fold change) downregulated by 6bE5 in keratinocytes. Around 600 genes were downregulated (>1.5 fold in each array, *p* < 0.01) in 6bE5‐expressing cells as compared to LXSN‐expressing cells.			
Primary sequence name	Accession #	*p* Value	Fold change
C1orf135	NM_024037	0.00	−13.90
C9orf94	NM_152702	0.01	−12.87
CDKN2C	NM_001262	0.00	−11.95
XPOT	NM_007235	0.00	−10.38
NRG1	NM_013962	0.00	−9.99
LETM2	NM_144652	0.00	−9.86
KRR1	NM_007043	0.00	−9.75
BM850706	BM850706	0.00	−9.66
C12orf24	NM_013300	0.00	−9.62
RRM2	NM_001034	0.00	−9.45
EIF2S1	NM_004094	0.00	−9.42
KIAA0114	CR611723	0.00	−9.25
WIBG	BC009627	0.00	−9.24
PRR15	NM_175887	0.00	−8.96
LBR	AJ381562	0.00	−8.84
FAM54A	NM_138419	0.00	−8.54
PTPN2	NM_002828	0.01	−8.53
TOP2A	NM_001067	0.00	−8.42
ANLN	NM_018685	0.00	−8.35
DNAH11	NM_003777	0.00	−8.34
MED26	BC030138	0.00	−8.28
THC2272132	THC2272132	0.00	−8.10
SPC25	NM_020675	0.00	−7.87
LRP8	NM_033300	0.00	−7.80
CCBL2	NM_019610	0.00	−7.76

**(B) Top 25 genes (by fold change) upregulated by 6bE5 in keratinocytes. Around 160 genes were upregulated (>1.5 fold in each array, *p* < 0.01) in 6bE5‐expressing cells as compared to LXSN‐expressing cells**.			
Primary sequence name	Accession #	*p* Value	Fold change
TNFSF10	NM_003810	0.00	3.97
FER1L4	NR_001442	0.00	4.10
CTSS	NM_004079	0.00	4.20
TNFSF10	BM978417	0.00	4.29
C3	NM‐000064	0.00	4.31
SOD2	AA970543	0.00	4.32
GPNMB	NM_001005340	0.00	4.41
LTB	NM_002341	0.00	4.87
KYNU	NM_003937	0.00	5.01
DCN	NM_001920	0.00	5.30
LOX	NM_002317	0.00	5.36
PGLYRP4	NM_020393	0.00	5.60
TNFSF10	NM_000594	0.00	5.76
C10ORF10	NM_007021	0.00	6.11
FBXO32	NM_058229	0.00	6.55
YPEL4	NM_145008	0.00	6.68
MMP9	NM_004994	0.00	8.53
LOC387763	BC052560	0.00	8.78
PLA2G4C	NM_003706	0.00	8.79
MB2	AF100640	0.00	9.58
ICAM1	NM_000201	0.00	10.55
SERPINA3	NM_001085	0.00	11.93
LOC57400	AF264627	0.00	49.09
LOC57398	AF264621	0.00	105.05
drug‐sensitive protein 1	AY227436	0.00	326.09

**(C) Genes were classified according to enriched gene ontology terms**.			
**GO Class ID**	**Definitions**	**Counts**	**Fractions**
GO:0008152	Metabolism	42	17.10%
GO:0007049	Cell cycle	33	13.40%
GO:0006139	Nucleobase, nucleoside, nucleotide and nucleic acid metablism	30	12.20%
GO:0016043	Cell organization and biogenesis	22	8.90%
GO:0006996	Organelle organization and biogenesis	19	7.70%
GO:0006259	DNA metabolism	16	6.50%
GO:0006950	Response to stress	15	6.10%
GO:0009058	Biosynthesis	7	2.90%
GO:0006810	Transport	7	2.90%
GO:0007275	Development	7	2.90%
GO:0009605	Response to external stimulus	5	2.00%
GO:0007165	Signal transduction	5	2.00%
GO:0030154	Cell differentiation	5	2.00%
GO:0007154	Cell communicaton	5	2.00%
GO:0007010	Cytoskeleton organization and biogenesis	5	2.00%
GO:0000003	Reproduction	4	1.60%
GO:0019725	Cell homestasis	3	1.20%
GO:0009653	Morphogenesis	3	1.20%
GO:0009056	Catabolism	3	1.20%
GO:0008283	Cell proleferation	2	0.80%
GO:0006519	Amino acid and derivative metabolism	2	0.80%
GO:0006811	Ion transport	2	0.40%
GO:0015031	Protein transport	1	0.40%
GO:0006464	Protein modification	1	0.40%
GO:0019538	Protein metablism	1	0.40%
GO:0006350	Transcription	1	0.40%
**Total**		**246**	**100.00%**

*Note*: Microarray was performed on three different donor pools of primary foreskin keratinocytes which were stably transduced with 6bE5 or LXSN. Dye swap was performed for each replicate. (A) Top 25 genes (by fold change) downregulated by 6bE5 in keratinocytes. Around 600 genes were downregulated (>1.5 fold in each array, *p* < 0.01) in 6bE5‐expressing cells as compared to LXSN‐expressing cells. (B) Top 25 genes (by fold change) upregulated by 6bE5 in keratinocytes. Around 160 genes were upregulated (>1.5 fold in each array, *p* < 0.01) in 6bE5‐expressing cells as compared to LXSN‐expressing cells. (C) Genes were classified according to enriched gene ontology terms. Significantly represented (*p* < 0.01) biological processes are shown.

**Figure 2:**
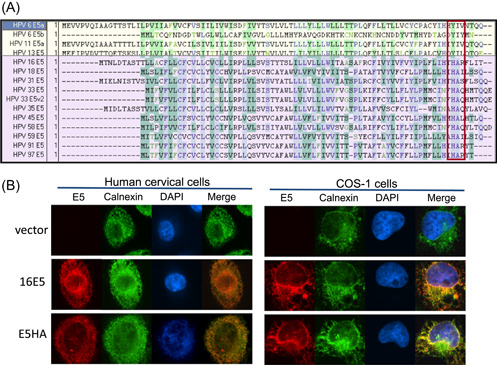
Construction of the HPV‐16 E5 mutant, H77YA78I, 16HA (A) Alignment of low‐risk (yellow) and high‐risk (purple) HPV E5 protein sequences. Mutations were made in boxed region. Amino acids histidine and alanine (highly conserved in high‐risk HPVs) were swapped for tyrosine and isoleucine (highly conserved in low‐risk HPVs). (B) Mutant co‐localization with the ER‐marker calnexin (green) in stably transduced HECs and transfected COS‐1 cells. AU1‐tagged E5 is visualized by an anti‐AU1 antibody (red). DAPI (blue) is the nuclear stain. DAPI, 4′,6‐diamidino‐2‐phenylindole; HPV, human papillomavirus

We next attempted to define the protein domain that might account for the biological differences between the low‐ and high‐risk E5 proteins. An alignment of the E5 amino acid sequences from several low and high‐risk HPV types was performed (Figure 2A). Of note are amino acids at position 77 and 78 in HPV‐16 E5. These two residues, histidine and alanine, are highly conserved in E5 proteins from all alpha high‐risk HPVs. However, in low‐risk HPVs, a tyrosine and isoleucine are conserved at the same positions. Based on this observation, we generated the 16E5 mutant H77YA78I (16HA) (Celtek Biosciences), in which the histidine and alanine residues (conserved in high‐risk HPVs) were replaced with tyrosine and isoleucine (conserved in low‐risk HPVs). Immunofluorescence was used to confirm the expression and localization pattern of the mutant construct, which merged with the ER‐marker calnexin in stably‐expressing primary human cells and transfected COS‐1 cells (Figure 2B).^46^ Our previous study found that this 16HA was unable to repress COX‐1 mRNA and XBP‐1 splicing in primary keratinocytes.^46^ This pattern is similar to the previously published localization of the wild‐type 16E5 protein^47^ and low‐risk 6E5.^35^

**Figure 3:**
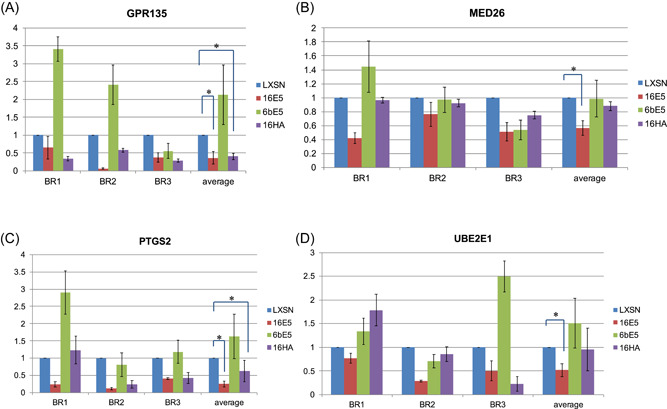
Real‐time RT‐PCR confirmation of genes in 16E5 arrays. Four genes altered by 16E5 in the microarray analysis were chosen for confirmation by real‐time RT‐PCR (A–D). 6bE5 did not cause significant changes in expression of these genes. In addition, 16E5 mutant 16HA is also defective for a reduction in these genes. Three biological replicates (BR) were tested for each gene. Data for all experiments are normalized to GAPDH. *n* = 3. Bars represent means ± SEM. *Indicates *p* value < 0.05 as determined by a paired student's *t* test. RT‐PCR, reverse‐transcription polymerase chain reaction; SEM, standard error of mean

**Figure 4:**
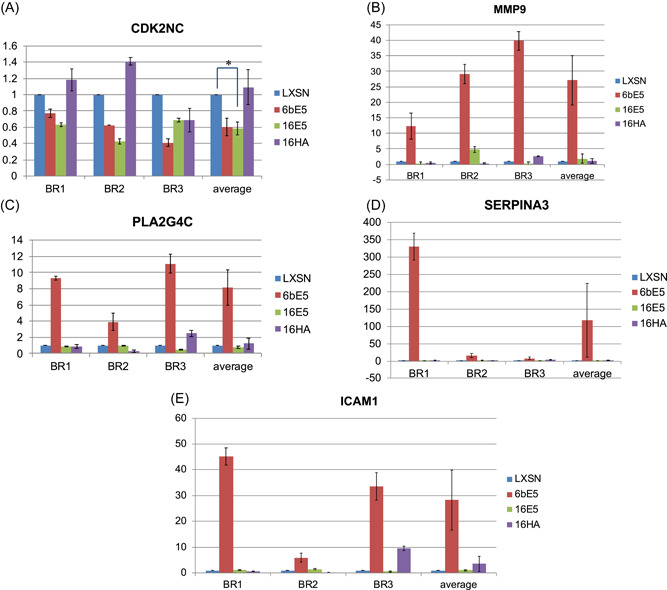
Real‐time RT‐PCR confirmation of genes in 6bE5 arrays. Five genes of the ~720 genes changed in 6bE5‐transduced cells were chosen for real‐time confirmation (A–F). Three biological replicates (BR) were tested for each gene. Data for all experiments are normalized to GAPDH. *n* = 3. Bars represent means ± SEM. *Indicates *p* value < 0.05 as determined by a paired student's *t* test. RT‐PCR, reverse‐transcription polymerase chain reaction; SEM, standard error of mean

The mutant 16HA was then included in real‐time PCR confirmation of the microarrays. Genes chosen for confirmation were chosen based mainly on fold change, available literature, and relevance to cancer. Real‐time RT‐PCR was used to confirm the downregulation of four genes affected by 16E5 (Figure 3). All four genes were downregulated; however, only two were statistically significant. Interestingly, three of these genes were not altered by either the 6bE5 or the 16HA mutant, suggesting that the ability of the high‐risk E5 protein to downregulate these genes may be dependent upon two highly‐conserved C‐terminal amino acids. In addition, five genes affected by 6bE5 in the microarray were selected and confirmed by real‐time PCR (Figure 4). However, similar to the wild‐type 16E5, the 16HA mutant failed to induce gene expression changes in a similar manner to 6bE5 (Figure 4). This suggests that the two C‐terminal amino acids conserved in low‐risk HPVs are not sufficient to confer properties of the low‐risk 6bE5 when introduced in isolation into a 16E5 sequence.

In brief, our results with primary keratinocytes differ very significantly from those obtained in the previously published HaCat cell study,^19^ both in terms of the number and types of genes that were altered in expression. That study found that 179 genes were significantly altered (no fold change cutoff, *p* < 0.01) by 16E5 expression, including lamin A/C, PKC‐γ, and PI3K. 16E5 was suggested to inhibit apoptosis by affecting pathways involved in cell adhesion, motility, and mitogenic signaling. In contrast, our analysis indicated that a far smaller subset of genes (~25) were consistently affected (fold change >1.5, *p*‐value < 0.01) in three independent preparations of primary keratinocytes. Most of these genes are involved with metabolism or biosynthesis pathways. The difference between our data and the above study may be due to site origins or genetic background (foreskin vs. adult trunk keratinocytes), cell status (primary vs. immortalized), or gene expression level (stable expression vs. transient inducible expression).

In addition, our data indicate that while the gene expression changes induced by 16E5 are subtle, 6bE5 is able to induce a far greater subset of gene changes, both in terms of number and fold change of affected genes. The difference in the profile of gene expression altered by 6bE5 and 16E5 is likely to result in different biological functions of the E5 proteins from high‐ and low‐risk HPVs. For example, it is interesting to note that in our findings, 16E5 significantly downregulated PTGS2/COX‐2 by microarray and real‐time PCR. However, 6bE5 showed no significant change in this gene by microarray, and if at all, demonstrated increased PTGS2/COX‐2 levels by real‐time PCR. In fact, previous studies have demonstrated increased levels of COX‐2/PTGS2 in recurrent respiratory papillomatosis (RRP) lesions, which are caused by low‐risk HPV 6b and 11.^48^, ^49^ It is possible that 6bE5 contributes to the increase of COX‐2 levels seen in low‐risk HPV infection. Our findings suggest that differences in gene expression altered by low‐ versus high‐risk E5s may contribute to the differences in pathology resulting from infection by low‐ versus high‐risk papillomaviruses.

## CONFLICTS OF INTEREST

The authors declare no conflicts of interest.

## Supporting information

Supporting information.

Supporting information.

Supporting information.

Supporting information.

## Data Availability

The data that supports the findings of this study are available in the supplementary material of this article.
